# Current Understanding of Natural Antibodies and Exploring the Possibilities of Modulation Using Veterinary Models. A Review

**DOI:** 10.3389/fimmu.2020.02139

**Published:** 2020-09-10

**Authors:** G. IJsbrand Reyneveld, Huub F. J. Savelkoul, Henk K. Parmentier

**Affiliations:** ^1^Faculty of Science, VU University, Amsterdam, Netherlands; ^2^Adaptation Physiology Group, Department of Animal Sciences, Wageningen University, Wageningen, Netherlands; ^3^Cell Biology and Immunology Group, Department of Animal Sciences, Wageningen University, Wageningen, Netherlands

**Keywords:** natural (auto-)antibodies, disorders, food animals, modulation, homeostasis

## Abstract

Natural antibodies (NAb) are defined as germline encoded immunoglobulins found in individuals without (known) prior antigenic experience. NAb bind exogenous (e.g., bacterial) and self-components and have been found in every vertebrate species tested. NAb likely act as a first-line immune defense against infections. A large part of NAb, so called natural autoantibodies (NAAb) bind to and clear (self) neo-epitopes, apoptotic, and necrotic cells. Such self-binding antibodies cannot, however, be considered as pathogenic autoantibodies in the classical sense. IgM and IgG NAb and NAAb and their implications in health and disease are relatively well-described in humans and mice. NAb are present in veterinary (and wildlife) species, but their relation with diseases and disorders in veterinary species are much less known. Also, there is little known of IgA NAb. IgA is the most abundant immunoglobulin with essential pro-inflammatory and homeostatic properties urging for more research on the importance of IgA NAb. Since NAb in humans were indicated to fulfill important functions in health and disease, their role in health of veterinary species should be investigated more often. Furthermore, it is unknown whether levels of NAb-isotypes and/or idiotypes can and should be modulated. Veterinary species as models of choice fill in a niche between mice and (non-human) primates, and the study of NAb in veterinary species may provide valuable new insights that will likely improve health management. Below, examples of the involvement of NAb in several diseases in mostly humans are shown. Possibilities of intravenous immunoglobulin administration, targeted immunotherapy, immunization, diet, and genetic modulation are discussed, all of which could be well-studied using animal models. Arguments are given why veterinary immunology should obtain inspiration from human studies and why human immunology would benefit from veterinary models. Within the One *Health* concept, findings from veterinary (and wildlife) studies can be related to human studies and *vice versa* so that both fields will mutually benefit. This will lead to a better understanding of NAb: their origin, activation mechanisms, and their implications in health and disease, and will lead to novel health management strategies for both human and veterinary species.

## Introduction

Natural antibodies (NAb) are defined as immunoglobulins found in individuals without (known) prior antigenic experience (1). Albeit a heterogeneous group, NAb are generally characterized as oligo-specific low affinity binding immunoglobulins which recognize exogenous and self-antigens (2). The majority of reported NAb are IgM and IgG, whereas IgA is much less studied and described. NAb have germline encoded VH and VL regions that restrict their binding capacity to phylogenetically conserved epitopes (3), in contrast to adaptive immunoglobulins that could theoretically recognize any epitope of an antigen. NAb have minimal N-nucleotide insertions and few or no somatic hyper-mutations and therefore are of low affinity (4). In comparison, low affinity NAb have a dissociation constant (K_d_) ranging between 10^−4^ and 10^−6^ M, whereas high affinity conventional antibodies range between 10^−6^ and 10^−10^ M (5). With respect to their functions, NAb were reported to initiate apoptosis (6), enhance T cell proliferation (7), activate complement (8–10), opsonize antigen (11), enhance antigenicity (12), target antigen to lymph nodes (13), and are involved in FcR-mediated phagocytosis (10). They also act as broad neutralizing agents (6) and endogenous adjuvants for CD8+ T-cell responses (14), and they sustain differentiation and maturation of dendritic cells (15, 16) (Table 1). For extensive reviews of NAb functions see also references 4 and 17.

**Table 1:** Involvement of natural antibodies in immune responses and immune status.

	**References**
Initiation of apoptosis	(6, 17)
Complement activation	(8–10, 18)
FcR-mediated phagocytosis	(10, 19)
Neutralization of infective agents	(6, 18, 20, 21)
Adjuvanting properties	(14, 22)
Maturation of dendritic cells	(15, 16)
Clearance of senescent/necrotic cells	(23, 24)
Prevention of autoimmunity	(17, 25–28)
Opsonization of antigens	(11)
Enhancement of antigenicity	(12)
Antigen targeting to lymph nodes	(13, 18)
T cell proliferation	(29)
Allograft rejection	(30)

A substantial part of NAb can react with intracellular and membrane expressed autoantigens and circulating macromolecules and haptens that are conserved during evolution. Such antibodies are called natural autoantibodies (NAAb) (23, 24). NAAb can react with many autoantigens, and damaged and senescent cells. Damage or senescence of cells might be due to oxidative mechanisms resulting in the generation of neo-epitopes on or within the cell. Thereby, NAAb facilitate antigen-mediated removal of apoptotic cells by phagocytosis and display anti-inflammatory activity. This decreased exposure to intracellular autoantigens from apoptotic cells might also mitigate the development of autoimmune diseases (17, 25). On the other hand, NAb are indicated in the pathogenesis of autoimmunity, inflammatory bowel diseases, contact hypersensitivity, and sepsis (31), but only a minority of NAb and NAAb have pathogenic features (29). Moreover, many individuals possess antibodies directed against common epitopes in highly mutating viral infections, like influenza and HIV. These, so-called “broadly neutralizing antibodies” share some characteristics with NAb (20, 21). Antibodies binding previous versions of the viral strain consist of about 0.01% of the antibodies raised after infection or vaccination and react with all variants of the virus and thus appear to be multi-specific. Such antibodies might constitute passive vaccines against non-mutable common structures in otherwise highly mutating viruses.

Since their initial discovery early 1960s, NAb were found in every vertebrate species investigated: mammals (2), birds (32, 33), fish (34, 35), and reptiles (36). Nevertheless, NAb have been regarded as contradictive with established immunological dogmas, but gradually receive more attention in main stream immunology.

## B1-Cells Are the Predominant Source of Natural Antibodies

The origin of NAb has mostly been studied in mice, where they predominantly originate from B1-cells (B220low, CD19high, IgMhigh, CD23–, CD43+), which are further delineated in B1a-cells (CD5+) and B1b-cells (CD5–). B1-cells are present within peritoneal and pleural cavities and lymphoid tissues like spleen and lymph nodes (37). Such B1-cells were found to be long-lived and retain their self-renewing capacity and hence their suggested innate-like properties. Besides their reduced junctional diversity and their low somatic hypermutation, their IgH VH gene rearrangements favor usage of the V_H_12 segment generating antibodies able to react with phosphatidylcholine. Phosphatidylcholine is a major lipid in general the protective mucus layer of the gastrointestinal tract and membranes of various bacterial species. These B1-cells maintain an active first line of defense against bacteria (37). The typical V_H_12 containing B1 receptor is able to reprogram B2-cells into becoming B1-cells and thereby adopting the B1 receptor and other B1-cell surface markers and start to spontaneously produce antibodies. Therefore, apparently no distinct progenitor cells for B1-cells required. This shows that driving the generation of B1-cells is because of their special B-cell receptors (38).

Approximately 90% of NAb in mice are secreted by B1a-cells whereas B1b-cells and marginal zone (MZ) B-cells do so to a lesser extent (2). Approximately 80% of total murine serum IgM is derived from B1-cells under steady state conditions (17). Therefore, B1-cells were regarded as the main source of NAb whereas B2-cells (B220+, CD19+, IgMlow, CD23+, CD43–) are considered as the main source of conventional antibodies. In humans B1-cells were defined as CD20+CD27+CD43+CD70– (39), and CD19+CD20+CD27+CD38low/intCD43+. The latter cells were found to decrease with aging, probably because of poor bone marrow production which might have an impact on the ability to fight infections and the development of age-related diseases (40). In all other species, B1-cells require identification and characterization, and their role in the release of NAb is unknown. For instance, B1-cells in cattle were defined by the originally used murine markers CD5 and CD11b and subdivided in CD5+CD11b- B1a-cells, CD5-CD11b+ B1b-cells and CD5-CD11b- conventional B2-cells (41). Flow cytometry analysis showed a distinct cell population of IgM+, pSYK+ cells, indicating B1-cells in dairy cattle (42). Phenotypical properties of NAb-secreting B-cells in other species remain enigmatic.

Although NAb B-cells are regarded as pre-defined, it is suggested that a NAb B-cell still requires antigenic selection and even T-cell help, remarkably by yδ T-cells (29), but the exact mechanisms are not known (2). One theory suggests that B1-cells are educated at mucosal (intestinal) sites under the influence of the microbiome. This is supported by the finding that NAb binding the carbohydrate Galα1-3Galβ1-4GlcNAc (α-Gal) in GALT^−/−^ mice were influenced by the *Clostridiales, Bacteriodales, Lactobacillales*, and *Deferribacterales* orders (43). Anti-Gal NAb can block the entry and transmission of membrane-binding viruses as these cannot produce glycosylated proteins themselves (44).

Fetal and neonatal self-reactive B1-cells do not show clonal expansion upon B-cell receptor (BCR)-signaling because of the expression of the inhibitor CD5 and a lack of fully functional CD19. Consequently, these B1-cells are silenced and thereby prevented to induce autoimmunity. Nevertheless, B1-cells can respond rapidly to different infections by firstly migrate to secondary lymphoid tissues and subsequently differentiate into IgM-secreting cells (45). Thus, stimulation of murine B1-cells in peritoneal cavities does not directly lead to the secretion of NAb as these activated B1-cells migrate toward the spleen and lymph nodes before the secretion of natural IgM takes place (46, 47). However, by Toll like receptor (TLR)-mediated activation these B1-cells can respond and circumvent the BCR-induced signaling block (45). The restricted fetal preimmune repertoire in humans may contain potentially beneficial self-reactive innate-like B cell specificities that are involved in the removal of apoptotic cells and shaping of the gut microbiota after birth (48). Another hypothesis is that IgM NAb B-cells are educated by maternal IgG, which in humans is the only antibody isotype that passes the placental wall. This IgG pool represents the unique environment experienced by the mother and is passed into the neonate as a single passive immunization. This idea is supported by observations that human neonates share a similar IgM profile with each other, whereas the IgG profiles of neonates are similar with their respective mothers (49). During aging, the IgM and IgG profiles merge suggesting that the IgM repertoire is shaped by maternal IgG. Therefore, maternal IgG may act as the immunological homunculus (50) shaping or educating the neonatal immune system. Whether this is true for all species is currently unknown. Bovine calves that do not receive maternal antibodies prior to intake of colostrum showed both IgM and IgG self-binding antibodies (51), which are, however, dramatically increased after colostrum intake. Nevertheless, the exact origin of germline encoded NAb remains unknown and requires further investigation.

## The Mechanisms Leading to Natural Antibody Secretion Are Not Fully Understood

Little is known about the mechanisms that underlie the secretion of NAb, but Holodick et al. (2) propose some interesting models that may explain the activation routes of NAb B-cells. The first model states that a NAb B-cell is pre-existing, but in order to secrete NAb it must undergo classical maturation, activation and differentiation into plasma cells and memory B-cells. The existence of homeostatic self-binding NAb B-cells in this model could then be explained by the fact that IgM-BCRs have similar low affinity binding like IgM NAb and would therefore be able to escape negative selection. However, the model does not explain the necessity of structurally and functionally unique pre-existing immunoglobulins if editing and selection procedures will take place eventually.

The second model embraces the idea that a NAb B-cell is pre-existing and generates NAb at a constant rate without the need for antigenic activation. This is supported by the observation that NAb are universally present in many species without (known) antigenic stimulation and that IgM levels seem constant throughout life (52), suggesting that NAb are a tightly regulated pool of immunoglobulins. However, the model fails to explain the presence of IgG and IgA NAb (53, 54) as it does not allow hypermutation and class switching to occur. Instead, an antigenic overload would require compensation by adaptive IgG's that could lead to an excessive or irrelevant immune response.

The third model suggests that a NAb B-cell is pre-existing but that a slight antigenic push is required in order to secrete NAb. While the secretion of NAb has been implicated to be T-cell and antigen independent, there is a possibility that exogenous antigens are indeed involved in B-cell activation, but in a B-cell Receptor (BCR) independent manner instead of an antigen independent manner. Besides BCR, B-cells also express innate receptors (e.g., TLRs), and it was demonstrated that they are important mediators of B-cell activation, proliferation, and class-switching (45, 55). One example is the BCR independent secretion of natural and self-reactive immunoglobulins binding LPS (55), suggesting the involvement of the LPS recognizing TLR4. Moreover, B1-cells from naïve mice stimulated with IL-5 and TLR-agonists secreted IgM against oxidized lipids *ex vivo* (3, 56), further suggesting that the secretion of NAb is BCR independent and rather regulated by innate pathways. Recently, it was also demonstrated that TLRs are critical for regulating antibody production by B1a-cells (45, 57). Microbial-sensing TLR (e.g., TLR2 and TLR4) are required for anti-microbiota B1a-cell responses, whereas nucleic-acid binding TLR7 and TLR9 control B1a-cell responses to self-antigens like phosphorylcholine (the headgroup of oxidated phosphatedylcholine) and microbiota-derived antigens (57). Unfortunately, this model is not able to explain the constant secretion of IgM as it will only provide IgM when there is an antigenic demand.

The fourth model tries to create a middle ground by stating that a NAb B-cell is pre-existing and secretes IgM NAb in steady state conditions. However, it is able to differentiate into IgG or IgA secreting plasma cells after antigenic stimulation that allows somatic hypermutation and class switching. This view is supported by the finding that IgG NAb against citrate synthase (CS) in the pericardial fluid (PF) correlated with antibody titers against pathogens associated with cardiovascular diseases, whereas anti-CS IgM NAb were not (58, 59). This also implies that only IgM antibodies could be defined as NAb according to the classical definition. As opposite to classical antigen-induced B-cell responses which are helped by αβ T-cells, NAb producing B cells were indicated not to require cognate T-cell help but depend on soluble mediators produced by γδ T-cells which should play a prominent role in their regulation through the fine-tuning of IL-4-levels (29). The earlier arising γδ T-cells during ontogeny should be better positioned than αβ T-cells to shape the developing repertoire of NAb. Since ligand specificities of NAb and γδ T-cell receptors appear to overlap, this may allow γδ T-cell help for certain NAb specificities (29). Lastly, since vertebrates share many macromolecules with the microbiome, “cross reactivity,” and the role of the microbiome in shaping and maintaining the NAb-repertoires cannot be excluded. Many “classical” NAb may be initiated by the intestinal and oral cavity microflora (44, 60). In conclusion, further research regardless of species is required to fully understand the origin, induction and activation pathways of NAb B-cells and NAb.

## Natural Antibody Reactivity

Since their discovery in the early 1960s, NAb were neglected or denied within the immunological society because of their apparent contradiction with established immunological dogma's. Germline encoded immunoglobulins do not fit in the fundamentals of random VDJ-rearrangement, and the existence of self-binding NAb is incompatible with Burnet's clonal selection theory (61), stating that self-binding B-cells are selectively removed from the circulation. Furthermore, the properties of NAb could also be perceived as redundant because high-affinity binding and mono-specificity are regarded as key characteristics of relevant and effective immunoglobulins.

## Natural Antibodies Binding to Self-Antigens Act as Homeostatic Agents

On average, humans possess around 5 l of blood containing 4 x 10^9^ white blood cells per liter of blood of which 5% is comprised of B-cells. In turn, ~5% of the B-cell population are considered to be B1-cells, amounting to 5 x 10^7^ B1-cells in an average human which suggests that NAb are a major part of the systemic antibody pool (5, 62).

Autoantibodies have a bad reputation in immunology as they are the primary mediators in many autoimmune diseases. The majority of these disorders are hallmarked by the presence of autoantibodies against specific target antigens (63). For example, Graves' disease is characterized by antibodies targeting the Thyroid Stimulating Hormone (TSH) receptor, which results in an unregulated secretion of thyroid hormones (63). Autoantibodies against Ro/SSa and La/SSb are hallmarks of Sjögrens Syndrome, which is an autoimmune disorder that mainly affects mucous membranes and moisture-secreting glands in the eyes and mouth (64). More than 180 unique autoantibodies were identified in Systemic Lupus Erythematosus (SLE), a systemic autoimmune disease that affects multiple organs (65). Despite these negative associations, self-binding immunoglobulins can already be detected in future patients with autoimmune diseases years before the onset of autoimmunity without showing any signs of pathology (66, 67). A large portion of B-cells are self-binding under steady state conditions and murine B1a-cells are positively selected for self-reactivity (37). Moreover, 75% of early immature naïve murine B-cells and 20% of mature naïve B-cells are self-binding and somatic hypermutation even restores self-reactivity back to approximately 45% (68, 69). Despite the immense pool of diverse antigens available, “only” 100 immune diseases are known, of which half of them have signature antigens for autoantibodies, which is a very small part of the total proteome (63) as already indicated above (29). This raises questions about the nature of these hallmark antigens and why the rest of the proteome is not a trigger for autoimmunity.

In all “normal” healthy individuals, in human cord blood and in “antigen-free” mice (1), self-binding antibodies are found of the IgM, IgG, and IgA classes, binding a variety of structurally different serum proteins, surface molecules, and intracellular structures like ubiquitin, collagen, hemoglobin-α, ss- and dsDNA, fibrin, the carbohydrate α-Gal, extracellular cytokines (54), nuclear membrane antigens (70) and cell membrane components such as oxidized lipoproteins (24, 71). Exposure of these kind of self-antigens in the wrong context, for example due to necrosis or aberrant apoptosis could lead to unwanted presentation to adaptive immunity and subsequent autoimmunity with severe consequences for the affected individual. Therefore, it can be hypothesized that NAb neutralize these antigens before an adaptive immune response or inflammation is initiated against them. For instance, protective natural IgM's binding phosphorylcholine were negatively correlated with IL-6 and TH17 responses in SLE patients and could be related to the intestinal microbiota (72).

The antigens that are targeted by self-binding NAb may in fact function as Damage/Danger Associated Molecular Patterns (DAMPs), which are endogenous compounds that are constitutively expressed in all tissues. When released into the periphery during degranulation, cell injury or necrosis, they induce chemotaxis and various forms of immune activation (73). Heat shock proteins (HSP), annexin, S100 proteins and galectins are considered as signature DAMPS (74), but were also found to be targets for NAb (54). It was demonstrated that a pool of IgM's inhibited TLR mediated cytokine expression and mitogen activated protein (MAP) kinase activation *in vitro* and specifically induced inhibitory signaling pathways in innate immune cells (17). While this shows that NAb can have a direct inhibitory function on immune cells, it can be hypothesized that this is mediated by the formation of immune complexes, presumably with DAMPs. This would be an essential mechanism as decreased NAb-levels would leave DAMPs in the circulation and susceptible to be intercepted by adaptive immune cells, leading to a pro-inflammatory immune response. While this is beneficial in some cases, it could have severe consequences if inflammation occurred in the wrong context. Natural antibodies that are part of immune complexes can essentially be eliminated without the induction of inflammation, tissue repair and controlled catabolism.

Another mechanism that is suggested to be used by NAb against self-antigens is the regulation of B-cell development and selection by IgM's, as it was found that selective IgM deficient mice developed pathological autoimmunity (26). B cells, which express BCRs specific to hen egg lysozyme (HEL) were found to display diminished responsiveness to HEL stimulation in presence of soluble anti-HEL IgM antibodies suggesting IgM as negative regulator of BCR signaling. Soluble IgM antibodies may than act as decoy receptors for self-antigens that are recognized by membrane bound BCRs (75). Together with other data from FcμR^−/−^ mice, it was demonstrated that IgM NAb most likely facilitate the healthy development of B-cells in an FcμR-dependent manner (76, 77). As IgM is not able to pass the placental wall, an IgM-dependent IgM secreting B1-cell subset must pre-exist to facilitate this process (26). Natural IgM deficiency does affect B-cell development and selection and induces tolerance that prevents development of primary auto-immune diseases (26).

It is most likely that NAb also bind self-antigens that are not considered as typical DAMPs. For instance, antibodies binding many self-antigen fragments were found in liver from mice (78), liver, brain, kidney, and muscle from humans (79–83), and liver from cows (84) and poultry (85), but the functions of NAb binding such to be defined self-tissue antigens is still unknown. Hartman et al. (86) found that hybridomas from unmanipulated adult murine spleen cells revealed a pattern of a diverse VH usage reflecting the germline repertoire. The majority of murine organ reactive IgM NAb were polyreactive, expressing a broad range of unique and not indiscriminate reactivity patterns for both self and foreign antigens, suggesting that many naturally activated adult B-cells are highly polyreactive and that autoreactivity is a consequence of polyreactivity. The population of NAb exhibiting organ reactivity overlaps the populations of other IgM autoantibodies, and all these derive from a pool of polyreactive IgM antibodies which are polyclonally activated in the early immune response. These polyreactive natural antibodies may then represent a first line of defense and offer protection for the host against a variety of foreign agents (86).

In summary, it is very likely that self-binding NAb are systemic surveillance molecules that maintain immune homeostasis by aiding in the clearance of dying cells and apoptotic debris, thereby preventing activation of the immune system against the self and the subsequent development of self-immunity (3, 27, 28). In this light, it is fitting to regard pathological autoimmunity as a dysregulated state of initial homeostatic autoimmunity, rather than onset of previously absent self-recognition (87).

## Natural Antibodies Binding to Foreign Antigens Act as a First Line of Defense

Immunoglobulins in the absence of known immunization or vaccination against foreign antigens are persistently found in many species and have been isolated from various sources, including serum, milk, saliva, mucus, eggs, and feces. For an extensive review on NAb binding fungi, viruses and bacteria see also reference 17. NAb bind to foreign (microbial) antigens like lipopolysaccharide (LPS), lipoteichoic acid and peptidoglycans (88), which are present on many different types of bacteria. NAb were found to react with phosphorylcholine, which is present in the cell wall of *Streptococcus pneumoniae* (89), but also occurs on mammalian cell-membranes when phosphatidylcholine is oxidized. NAb are reactive with viruses and showed to bind to lymphocytic choriomeningitis virus (LCMV), vesicular stomatitis virus (VMV) (90), and various strains of Influenza (91). In addition, NAb also bind foreign (non-self) antigens that are not considered as pathological. Humans, rats, mice and alligators without previous immunization showed antibodies binding chicken red blood cells (92), whereas poultry (32), pigs (93), and cattle (94) all demonstrated to have NAb against Keyhole Limpet Hemocyanin (KLH). KLH is a large 390 kDa glycosylated protein from the gastropod *Megathura crenulata* which is found within the waters near California (95) and therefore an antigen that is highly unlikely to be experienced by non-marine individuals. To our knowledge, there is little evidence of cross reactivity with known infectious agents albeit the largeness of KLH does not completely exclude cross reactive antibodies. KLH is a potent immunogenic protein, but it does not cause adverse immune effects in humans and it is therefore a widely used vaccine carrier protein. Thus, KLH-binding NAb and likely NAb to other non-self-antigens appear to act as a vanguard of the immune system by protecting the host in an innate fashion during the relatively slow development of a specific antibody response.

## IgM and IgG Natural Antibodies in Health and Disease

IgM and IgG are the most extensively described classes of NAb in literature and were found to be implicated in many human infectious diseases and disorders, including neurological disorders, cancer, diabetes, and cardiovascular diseases. For an extensive review of the involvement of NAbs in health and various infectious-, tumor-, neurological-, and metabolic diseases see reference 94. Interestingly, lower levels of self-binding NAb are usually negatively correlated with disease onset and progress whereas high levels often correlate with protection or the absence of disease. In humans, profiles of NAb binding self-antigens were proposed as biomarkers or fingerprints for the physiological and health status of individuals (78, 96), including parasite infections such as malaria and schistosomiasis (97). The observed decline in the amount, or efficacy of homeostatic natural antibody levels were associated with a relative loss of protection against molecules involved in diseases whose incidence rises in the older age population, and that those individuals experiencing the greatest loss are at the highest risk. Natural antibodies were thus proposed as rheostats for susceptibility to several age-related diseases (98).

In veterinary species, clear relations between NAb and diseases were hardly expected, and thus much less studied, and are therefore much less known. In pigs, significant associations with osteochondrosis (OC) were found for IgM levels binding chondroitin sulfate A at 6 weeks of age (odds ratio (OR) 1.4 and 1.5), actin at 6 weeks of age (OR 1.4 and 1.3), thyroglobulin at 24 weeks of age (OR 1.5 and 1.3), and levels of IgG binding at 6 weeks of age (OR 1.7 and 1.4). Additionally, significant associations with OC were also found with IgM levels binding albumin or KLH at 6 weeks of age (OR 2.3 and 1.4), and with IgM levels binding actin at 24 weeks of age (OR 1.3) suggesting associations between the presence and levels of NAb-idiotypes at a young age and development of OC at later age (99).

## Natural Antibodies Against Brain Epitopes And Their Relationship With Neurodegeneration

For decades, the central nervous system (CNS) has been considered as an immune privileged site with relatively low to no detectable immune activity. Microglia and astrocytes can present antigen, but Major Histocompatibility Complex (MHC) -I and MHC-II expression is low and classical lymphatic drainage is apparently absent (100). However, the concept of an immune privileged brain has been moved aside in favor of “*an immunologically unique environment*” as it becomes more apparent that the CNS is more accessible to the immune system than has previously been thought. This access probably also applies for NAb as self-binding NAb targeting brain epitopes were found in healthy subjects or in the context of neurodegenerative disorders as exemplified below.

Multiple sclerosis (MS) is a neurodegenerative disorder in which an immune-mediated degeneration of myelin and subsequent loss of cognition is observed in about 50% of the patients. A human natural IgM (hIgM22) bound to oligodendrocytes in fresh brain slices (101). hIgM22 is thought to bind sulphated molecules, especially the myelin essential component sulfatide (102). Sulfatide acts as a negative feedback regulator for oligodendrocyte survival (103–105), which is the major cell type to produce myelin. A decrease in hIgM22 could lead toward neurodegeneration due to an increased availability of inhibitory sulfatide whereas binding of hIgM22 to sulfatide retains oligodendrocyte survival and subsequent myelin synthesis (102, 104). Indeed, hIgM22 induced remyelination in Theiler's Murine Encephalomyelitis Virus (TMEV), which is commonly used as a murine model of human MS. Lastly, hIgM22 was also able to bind to gangliosides and was therapeutic in a murine model of human Amyotrophic Lateral Sclerosis (ALS) (106).

Alzheimer's Disease (AD) is a neurodegenerative disorder characterized by deposition of Amyloid βeta (Aβ) plaques and Tau rich neurofibrillary tangles (NFT) (107). Aβ originates from the cleavage of the Amyloid Precursor Protein (APP), which is thought to play a role in synapse formation although its function is not fully understood (108). Neuroprotective NAb binding to assemblies of amyloidogenic peptides were reported to decrease with normal aging and advancing AD (109), and AD patients had decreased amounts of natural IgM and IgG against Aβ compared to age matched healthy individuals (110). This proved to be a therapeutic target of interest as APP-transgenic mice maintained their initial cognition level while having decreased cerebral Aβ depositions after intravenous administration of anti-Aβ antibodies (19). An observed side effect in mice, however, was the occurrence of cerebral microhaemorrhages. This was explained by a lower specificity of IgG for Aβ compared to IgM and the ability of IgG to pass the blood-brain barrier (111), demonstrating a more protective role of natural IgM in contrast to natural IgG. The second major AD associated protein is tau which, in its native form acts as a mediator in the generation and stabilization of microtubules. In NFT however, it is present in a hyper phosphorylated form (112) making it an excellent target for homeostatic immunity due to its modifications. Indeed, anti-tau IgG was found in healthy controls and pooled commercial IgG, although no clear differences in concentrations were found between these groups and AD patients (113). Of note, the therapeutic efficacy of anti-Tau antibodies was epitope dependent (114).

Many mouse strains suffer from an age-related progressive clustering of Periodic acid-Schiff granules within the hippocampus, which are characterized by the expression of a not fully defined carbohydrate neo-epitope. It was found that ICR-CD1, BALB/c and SAMP8 mouse strains have natural IgM's against these carbohydrate structures at all ages and even under germfree conditions (115). Strikingly, the same study also found that serum of rats, rabbits, goats and even commercially available antibodies also reacted with pathological granules in hippocampal tissue of ICR-CD1 mice, suggesting that they are conserved and widespread across species. In summary, literature (Table 2) demonstrates that brain epitopes are targets for NAb and that decreased levels can be negatively associated (or correlated) with neurodegeneration, whereas protection to behavioral disorders such as schizophrenia were correlated with for instance protective platelet associated autoantibodies (117).

**Table 2:** Involvement of natural antibodies in disorders.

**Species**	**Antigen(s)**	**Isotype**	**Disorder**	**Protection**	**References**
Human, mice	Various self-antigens and neo-epitopes	IgM and IgG	Various: anti-inflammation, OxLDL, tumors, neurologic diseases, infections, clearance senescent cells and cytokines, passive protection to autoimmunity and tissue injury	Yes	(116)
Pig	Chondroitin sulfate,	IgM, IgG	Osteochondrosis	Yes?	(99)
	Actin, KLH				
	Thyroglobulin				
Mice	Myelin sulfatide	IgM	Multiple sclerosis	Yes	(101)
Human	Amyloid	IgM	Alzheimer	Yes	(109–111)
mice	Amyloid beta-peptide	Polyclonal	Alzheimer	Yes	(19)
Human	Platelets	IgG	Schizophrenia	Yes	(117)
Human	Malondialdehyde	IgM	Schizophrenia	Yes	(118)
Pig	Myelin basic protein	IgM	unknown		(119)
Chicken	PC-BSA	IgM	Non-aggressive behavior		(120)
Chicken	PC-BSA	IgG	Aggressive behavior		(120)
Human	oxLDL	IgM	Carotid atherosclerosis	Yes	(121)
Human	PC-KLH, PC-BSA	IgG	Cardiovascular diseases	Yes	(122, 123)
Human	Phosphorylcholine,	IgM	Atherosclerosis,	Yes	(27, 124)
	Cardiolipin		Stroke,		
			Myocardial infections		
Mice	Malondialdehyde	IgM	Hepatic inflammation	Yes	(125)
Mice	Phosphoryl-enriched-	Not specified	Non-alcoholic	Yes	(126)
	Pneumococci		Steatohepatitis		
Mice	Oxidized phospholipids	IgM	Atherosclerosis	Yes	(127)
Mice	Phosphorylcholine, T15-idiotype	IgM	Vein graft atherosclerosis	Yes	(128)
Chicken	KLH	IgM	longevity		(129, 130)
Cow	KLH	IgM, IgG	Mastitis	Unknown	(131)
Human	Low density lipoprotein	IgM	Atherosclerosis	Yes	(132)
		IgG	Pro-atherosclerosis		
Mice	CNS-cells	IgM	Remyelination		(101)
Mice	Gangliosides	IgM	Amyotrophic		(108)
			Lateral sclerosis		
Human	Galα1-3Galβ1-GlcNAc	IgM, IgG,	Henoch-Schönlein purpura		(43, 44, 54, 133)
		IgA	IgA nephropathy		
			Crohn's disease		

NAb could also influence behavior, depression or anxiety, as these mental states were demonstrated to show immune alterations in general (134). Decreased levels of IgM NAb against oxidative stress epitopes like malondialdehyde and azelaic acid were found in deficit schizophrenia (118).

Veterinary models on the relations between NAb and neurological disorders are scarce. Pigs that were housed for 9 weeks in a straw embedded environment showed higher levels of IgM NAb binding myelin basic protein (MBP) compared to pigs kept in a barren environment (119), suggesting that the straw embedded environment has an enriching effect on the brain and either results in higher NAb-levels or prevents a decrease of these antibodies in barren environment kept pigs. Interestingly, in the barren kept pigs increasing levels of IgM binding MBP positively correlated with a decrease in viral PPRSV RNA levels (135) suggesting that high NAb-levels to a self-antigen enhanced resistance to PRRSV. The underlying mechanism remained unknown. Also recently, higher levels of IgG NAb were found in poultry strains bred for aggressive behavior, whereas the non-aggressive strain showed higher levels of IgM NAb (120). Further research is required to understand the relationship between NAb (isotypes) and behavior, but the current data suggest that self-binding antibodies protect against autoimmunity, chronic inflammation and necrosis which may underlie neurological disorders and misbehavior.

## Natural Antibodies Against Tumor-Associated Epitopes and Their Anti-Tumor Effects

Cellular transformation occurs in all types of cells and may lead to the development of tumors, albeit this is a relatively rare phenomenon compared to the high frequency of spontaneous mutations that occur in an individual (136). Immune processes are likely involved in clearing corrupted cells or components out of circulation. NAb may play an important part in this as nearly all monoclonal tumor targeting antibodies isolated from cancer patients so far were oligo-specific low affinity binding pentameric IgM's (137). Furthermore, natural IgM's to cancer associated autoantigens were detected up to 5 years before onset of breast cancer (138), suggesting their pre-existence but also providing diagnostic value as early biomarkers.

Carbohydrate structures are highly expressed on tumor cells and can be recognized by NAb (139). SC-1 is an isolated monoclonal IgM from a signet-ring cell carcinoma patient (140) and binds to a carbohydrate modified version of decay acceleration factor B (DAF/CD55), which is highly expressed on tumor cells and aids in immune evasion (140). SC-1 mediated crosslinking of DAF resulted in tumor-regression and apoptosis of stomach cancers without showing cross-reactivity with healthy tissue (121). PAM-1, a natural IgM isolated from a gastric carcinoma patient, binds to a carbohydrate modified isoform of cysteine rich fibroblast growth factor receptor (CFR-1), which is expressed on malignant cells but not on healthy tissue (141). PAT-SM6 is a natural IgM isolated from a gastric cancer patient (142) and binds to a glycosylated form of glucose-regulated protein 78 (GRP78), which is found on malignant cells but not on healthy tissue (143). PAT-SM6 was also found to induce apoptosis in multiple myeloma cells binding to the glycosylated form of GRP78 while leaving healthy tissue unharmed (144). Another study found that activation of peritoneal B1-cells with the C-type lectin agonist monophosphoryl lipid A (MPL) and the TLR agonist trehalose-6,6'-dicorynomicolate (TDCM) lead to increased production of IgM NAb in mice. These IgM's were targeted at carbohydrate antigens and suppressed tumor growth of peritoneal metastasis via the classical complement pathway (145). In summary, NAb are able to challenge tumors by recognizing tumor specific antigens, specifically those with carbohydrate modifications. Circulating autoantibodies in cancer patients had high specificity for glycoxidation modified histone H2A suggesting that glycoxidation of proteins and related autoantibodies could act as early biomarkers of cancer (146).

## Natural Antibodies Against Oxidized Lipids and Their Role in Cardiovascular Disease

Atherosclerosis is a chronic inflammatory disease that is characterized by the accumulation of apoptotic cells and oxidized lipids, specifically oxidized Low Density Lipoprotein (oxLDL) (147). It was demonstrated that oxLDL is an important target for NAb. In mice, around 30% of the IgM NAb bound to oxidized lipids, atherosclerotic lesions or apoptotic cells (71). Autoantibodies to oxLDL derived from “naïve” atherosclerotic mice shared complete genetic and structural identity with antibodies from the classic anti-phosphorylcholine B-cell clone, T15, which protects against common infectious pathogens, including pneumococci. S. pneumoniae immunized mice showed high circulating levels of oxLDL-specific IgM and persistent expansion of oxLDL-specific T15 IgM-secreting B cells, a decreased the extent of atherosclerosis (148) and blocked uptake of OxLDL by macrophages (127). High levels of IgM NAb against oxLDL were associated with protection against carotid atherosclerosis in hypertensive humans (149), but high levels of IgG binding LDL could be pro-artherosclerosis (132). NAb binding phosphorylcholine conjugated to BSA or KLH were decreased in patients with cardiovascular diseases and SLE and therefore proposed as potential protective factors (122, 123). NAb against other oxidation-specific epitopes have also been described, including those against malondialdehyde and 4-hydroxynonenal which were found in mice under pathogen free and germfree conditions (150). Immunoglobulins against phosphatidylserine and cardiolipin are generally associated with thrombosis, whereas immunoglobulins against their oxidized forms are associated with protection against atherosclerosis (27, 124). Natural IgM and IgG against citrate synthase (CS) were found in serum of healthy individuals and pericardial fluid (PF) of patients that went through open heart surgery (58, 59). CS is a highly conserved mitochondrial inner membrane enzyme involved in the citric acid cycle which occurs in nearly every cell, and especially in mitochondria-rich heart muscle cells. A relatively high number of B1-cells were present within PF and the prevalence of IgM NAb in PF was only half the amount of serum anti-CS IgM NAb in comparison to the total Ig levels that were four to eight times higher in serum (58). Together, the data suggests that NAb play an important role in the regulation or prevention of cardiovascular diseases (Table 2).

## Natural Antibodies and Their Role in Transplantation Immunology

NAb play an important role in transplantation immunology and allograft rejection (30). NAb against the oligosaccharide moieties of the ABO blood group system have been well-described and a mismatched blood transfusion leads to hyper acute transplantation rejection with severe clinical consequences. Graft B-cells infiltrate coronary arteries resulting in cardiac allograft vasculopathy (CAV), an accelerated form of coronary artery disease (CAD) limiting the long-term survival after cardiac transplantation (151). It was found that half of 100 B-cell clones isolated from three CAV cases showed oligo-reactivity toward apoptotic cells, dsDNA, cardiolipin, LPS and insulin (152). Renal proximal tubular epithelial cells are considered relatively susceptible to ischemia reperfusion injury, and this was mediated by IgM NAb via the classical complement pathway (153). Higher levels of IgG NAb binding apoptotic cells prior to kidney transplantation negatively correlated with graft loss, which was mediated by C4b complement deposition (154). A subsequent study also found that polyreactive IgG clones from two kidney transplant recipients were able to bind to Human Leukocyte Antigen (HLA) class I, albeit non-native denatured HLA (155). It can be postulated that an incorrect collection and transplantation of the organ would induce stress and subsequent antigen modification, therefore allowing homeostatic NAb to attack the neo-epitopes within the graft. Together, these studies demonstrate that graft-rejection should not strictly be attributed to monospecific immunoglobulins but probably rather to NAb, although the threshold for initiating this antibody-mediated rejection is unknown.

Apart from studies on pig tissues for human transplantation, transplantation studies are not a main topic in veterinary species and therefore knowledge on the role of NAb in these models is completely lacking.

## Natural Antibodies Against Pathogens and Infections

NAb were acknowledged as a first line of defense to infectious agents (29). IgM NAb might be involved in tuberculosis as a decrease in serum IgM levels against phospholipids is observed after intensive phase treatment, probably due to a decrease in bacterial burden (156). However, a decrease in IgM contrasts with the observation in other models where a decrease in NAb usually is a negative predictor for disease. An age-dependent decline in IgM NAb against pneumococcal capsular polysaccharides (PPS) and IgG NAb against a pool of virulence-associated proteins (VAP) of various *Streptococcus pneumoniae (S. pneumoniae)* strains was observed in humans, which could lead to increased susceptibility to *S. pneumoniae* infection (157).

In mice, NAb provide protection to viral infections (in an indirect fashion) by targeting virus-antibody complexes to the spleen and by contributing to the resolution of the acute phases of some viral diseases (18, 90). Infections are also prevented indirectly by NAb binding self-receptors such as CCR5, essential for the entry of the HIV virions (158). Maternal natural IgG antibodies protected neonatal mice from infection with enterotoxigenic *E. coli* infections when these antibodies were delivered across the placenta or through milk (159).

Protection to infections has been observed in veterinary species, but information is still scarce. High levels of NAb binding *Aeromonas salmonicida* protected goldfish against experimental infection (160), and high levels of NAb (and complement activity) correlated with fitness of wild boar when exposed to classical swine fever (161). Chickens bred for high levels of anti-KLH NAb showed improved resistance to avian pathogenic *E. coli* (162). The latter group also identified the existence of a single nucleotide polymorphism (SNP) variation in likely the TLR1A gene involved in determining the levels of natural antigen-specific IgM and total IgM antibodies in chickens (163). Heritability of natural IgM antibody levels was found which was absent or low for natural IgG or IgA antibodies (164).

In summary, NAb of the IgM and IgG class have been implicated in both health and diseases and are associated with protection against infections (Table 3) and disorders (Table 2) in humans and veterinary species. Future research should aim to expand this knowledge by further identifying more diseased states in which NAb are involved to further demonstrate their importance in maintaining health, and whether modulation of NAb-levels is feasible and desirable.

**Table 3:** Involvement of natural antibodies in prevention or combat of infection.

**Antigen(s)**	**Isotype**	**Infection**	**Effect**	**References**
Self- and microbiota	IgM, IgA	Microbiota	Protection	(37)
Glycans and microbiota	Not specified	Microbiota	Orchestration	(43)
Carbohydrates	IgM	Microbiota		(44)
LPS,KLH, Peptidoglycan	IgM, IgG, IgA	Unknown	Heritable	(88)
PC-rich	IgM	*S. pneumonia*	Protection	(89, 148)
*Streptococcus pneumonia*				
OxLDL, T15 idiotype				
VSV virus, Listeria	IgM	VSV virus, Listeria	Protection	(90)
Influenza strains	Not specified	Influenza	Protection	(91)
Malaria	IgM, IgG	Malaria	Protection	(97)
Schistosome		Schistosomiasis		
myelin basic protein	IgG	Porcine reproductive	Protection?	(119)
		respiratory syndrome virus		
Phospholipids	IgM	Tuberculosis	Protection?	(156)
Virulence-associated	IgM, IgG	*S. pneumonia*		(157)
Protein				
self-antigens,	IgM	Microbiota	Protective?	(18)
Phospholipids,				
T-cell independent-				
antigens				
CCR5	Not specified	HIV	Protective?	(158)
*Pantoea-1* microbes	IgG	*E. coli*	Protective	(159)
*Aeromonas salmonicida*	IgM	*A. salmonicida*	Protective	(160)
Chicken red blood cells	not specified	Classical swine fever	Protective?	(161)
KLH	IgM	*E. coli*	Protective	(162)
Phosphatidylcholine	IgG	*Plasmodium chabaudi*	Protective	(165)
Galα1-3Galβ1-GlcNAc	IgM, IgG, IgA	Block infections		(43, 44, 54, 155)

## IgA Natural Antibodies Require More Intensive Investigation

IgA is the most abundant immunoglobulin, with a production in humans of about 66 mg.kg^−1^.day^−1^ (reflecting 3–5 g per day). In humans, monomeric IgA (at 2 mg.ml^−1^) predominantly resides in serum where it functions as a potent pro-inflammatory agent by inducing rapid FcαRI mediated activation of neutrophils (166). In humans, two subclasses, IgA1 and IgA2, were identified in serum and secretions. These pro-inflammatory properties are not well-known as IgA has been perceived as a redundant non-inflammatory immunoglobulin in the intestinal lumen, which is true for secretory IgA (sIgA). sIgA originates at the basolateral side of mucosal areas where J-chain linked dimeric IgA (dIgA) is transported across the mucosal barrier into the lumen via the polymeric IgA receptor (pIgR). Upon its release into the lumen, dIgA retains a fraction of the pIgR, known as the secretory component (SC), which makes sIgA more robust and resistant against bacterial derived proteases. The SC also prevents association with the FcαRI which prevents interaction with immune cells, resulting in a homeostatic immunoglobulin that neutralizes microbiota and food antigens to prevent interactions with the host (167). Innate-like B1-cells can be stimulated by IL-5, IL-10, Toll-like receptor (TLR) agonists or whole bacteria to secrete IgM and IgA. As a pro-inflammatory immunoglobulin, serum IgA is crucial in the first line of defense against pathogens as a rapid activator of neutrophils. Meanwhile, homeostatic sIgA at mucosal sites most likely experiences the largest and most diverse amount of antigen interactions and is constantly challenged by this hostile environment.

IgA is perhaps the most important Ig-class, but the available literature on IgA NAb in humans and mice is lacking far behind in contrast to IgM and IgG NAb. Research in domesticated animals pointed to an important role for IgA NAb in binding larval antigens on mucosal tissues and aiding in the development of immunity to nematodes (168), and other parasites. Further studies into the role of IgA NAb in veterinary species are urgently needed.

## IgA Natural Antibodies in Serum Bind to Self-Antigens

An antigen microarray screening of self-binding NAb in serum and cord blood of ten mothers and their infants found IgA NAb against myelin oligodendrocyte glycoprotein (MOG), Gelsolin, Low Density Lipoprotein (LDL), Factor X and Protease in all subjects (54). While the reactivity of IgM NAb was nearly always higher than the reactivity of IgA NAb to a specific antigen, this was not the case for High Density Lipoprotein (HDL) and α2-microglobulin. On other occasions, IgA NAb showed higher reactivity against HDL, α2-microglobulin, LDL, Factor X, and Gelsolin compared to IgG NAb. Additional research is required to understand why IgA specifically seems to favor these antigens. Other studies found IgA NAb against α-Gal, which is considered as one of the most abundant natural antibodies (169). Anti α-Gal IgA was found in healthy subjects (170) but was also associated with Henoch-Schönlein purpura, IgA nephropathy and Crohn's disease (133). As serum IgA is a potent pro-inflammatory immunoglobulin, a sufficient amount of IgA NAb in serum against foreign antigens could be very beneficial in the critical time period of adaptive immunoglobulin development, by rapidly recruiting neutrophils to the side of infection. A pro-inflammatory response against self-antigens is questionable, unless it concerns oxidized, or (carbohydrate) modified (neo-epitope) forms of these antigens.

## IgA Natural Antibodies at Mucosal Sites Likely Originate From Committed B1b-Cells

Commensal gut bacteria are targeted in the small intestine by polyclonal oligo-specific B1b-cell derived IgA NAb whereas B1a-cells recognize restricted microbial regions (171). In contrast to B1a-cells, B1b-cells are able to switch to IgA+ plasma cells in a T-cell independent manner under the influence of TGF-β and Retinoic Acid (RA), which induces upregulation of α4β7+ and CCR9, providing a gut-homing phenotype (171, 172). A follow-up study found that naïve B-cells recirculated through Peyer's Patches to become IgA-secreting plasma cells in germfree and antigen-free mice (173). So it appears that B1b-cells are committed to eventually secrete IgA NAb at mucosal sites whereas activated B1a-cells migrate from the peritoneal cavities toward the spleen where they eventually secrete IgM (46, 47). Targeted modulation of the B1b-cell population might improve or diversify IgA NAb-responses at mucosal sites which could result in a better protection against exposure to microbes.

Chickens supplemented with probiotics showed higher levels of NAb (IgM and IgG) in their serum and intestines (IgA and IgG). These NAb also reacted with bacterial exotoxins (174). This implicates that studies on the role of microbes and hygiene in the formation of serum (IgM and IgG) and mucosal NAb (IgA) via dietary interventions could add in health management of both humans and veterinary species.

## IgA Natural Antibodies in Milk May Shape Natural Immunity of the Infant

Human milk is highly saturated with sIgA in concentrations up to 12 g/l in colostrum and 1 g/l in mature milk (175). These IgA NAb bind to endogenous antigens like actin, myosin, tubulin, transferrin, thyroglobulin, spectrin, laminin, myoglobulin, and native DNA (176, 177). Human colostrum derived sIgA reacted *in vitro* with human Hep-2 cells and monkey ovary, pancreas and adrenal gland tissue while in a lesser extend to monkey liver, testes, salivary gland, muscle, and thyroid glands (175). IgA NAb in milk can also be directed against foreign antigens, like protein disulfide isomerase (PDI) of *Toxoplasma gondii* (178). There is probably an interesting link between IgA NAb in milk and the gut. One study phenotyped milk derived B-cells as CD38-high, complement receptor-low, indicating that the milk derived B-cell population predominantly contained plasma blasts and plasma cells that actively secreted immunoglobulins. Further phenotyping revealed that the majority of milk derived B-cells were α4β7+ CD62L–, which are migration patterns similar to Gut Associated Lymphoid Tissue (GALT) B-cells (179). These findings lead to the hypothesis that an IgA NAb-profile of the environment is created in the maternal gut, specifically by B1b-cells that locally switch to IgA to create a highly promiscuous pool of immunoglobulins that react to both foreign and self-antigens. Human breast milk or raw cow's milk-derived immunomodulatory cytokines, like TGF-β2 and (very low levels of) IL-10, might upon consumption induce a regulatory environment in the gut which induces Regulatory T-cells and leading to the production of IgA and IgG4. Supplying sIgA NAb in breast milk can potentially enhance intestinal immunity in early life (180).

The exact effect and function of maternal sIgA for the infant is not known, but it fits in Jerne's idiotypic immune network theory where natural IgA would act as an educator of the infant's immune system (181). In this model, maternal sIgA (Ab1) is elicited against an environmental epitope in the mother and transferred toward the infant via the milk. In the infant, an anti-idiotypic immunoglobulin (Ab2) is generated against the maternally acquired Ab1. Subsequently, a third immunoglobulin mimicking the Ab1 BCR (Ab3) is generated against Ab2, which allows the infant to imprint this maternal immunoglobulin or BCR within its own repertoire. Previously mentioned findings in serum further support this idea (49), where it was observed that maternal IgG educated the neonatal independent IgM repertoire. However, the relationships between serum IgA levels and maternal IgG and/or neonatal IgM were not investigated. In mice, it was already shown that anti-idiotypic IgM antibodies specific for the IgA myeloma protein TEPC-15 (anti-phosphorylcholine) specificity, share similar or even identical idiotypes (182). In summary, natural IgA NAb or maternally derived natural antibodies may provide protection of the infant gut and be involved in maturation of the mucosal immune system.

In most veterinary species (e.g., cows and poultry) IgA is not the predominant maternal antibody as its role is fulfilled by IgG. Birds receive maternal IgG in the yolk, and are thus hatched with the maternal antibody repertoire, including self-binding antibodies (183). Calves, like most mammalian food animals, receive maternal IgG via colostrum including self-binding antibodies (51). Whether these maternal IgG antibodies shape the neonatal antibody repertoire as discussed above for man is currently unknown.

## Modulating Natural Antibodies and Therapeutic Opportunities

NAb are important as a first line of defense against pathogens and as homeostatic agents that inactivate or clean up potential dangerous self-antigens. Modulation or enhancement of NAb-levels and their diversity could lead to new therapeutic strategies and new insights into the usefulness of NAb. There is increasing knowledge of NAb in humans and their implications in health and disease, but studying intentional enhancement or decrease of NAb-levels in humans faces ethical objections because the effects and eventual risks are unknown, therefore urging the use of animal models. Mice are usually the first model of choice as they are economically affordable, easy to handle and share many parallels with human immunology. While mice have given many tremendous new insights into human immunology, there are also significant differences in immune development, activation mechanisms and immune response as mice and men are different in physiology, anatomy, size and lifespan (184, 185). Using non-human primates would be a logical alternative as they come closest to humans in genetics, physiology and behavior (186), but they are expensive and also require tight ethical regulations.

Alternative animal models that would fill a niche between mice and men are veterinary species like cattle, poultry, sheep and pigs which are not as tightly restricted by regulations and relatively economically affordable. In addition, contemporary agricultural practices require more knowledge on the maintenance or enhancement of health and welfare in veterinary species as well. Pigs are physiologically and anatomically close to humans, sharing similarities in cardiovascular systems, feeding (omnivorous) and skin composition (187, 188). Chickens being the most wide spread and most consumed veterinary species would also be interesting models as some major immunological breakthroughs in the past were achieved in chickens, including the principles of graft vs. host reactions and the delineation of the adaptive immune system into immunoglobulin secreting B-cells and cell-mediated immunity by T-cells (189).

Findings from veterinary species can be translated back to humans, but can also be applied within the field of veterinary immunology itself. Veterinary species are constantly challenged by bacteria, viruses, and parasites which not only has a major impact on animal welfare but also on the economy due to prevention and treatment costs, production losses and premature culling (190, 191). Diseases of bacterial nature are often treated with antibiotics, but the popularity of antibiotics has decreased due to risk of antibiotic resistance. Vaccination has received more popularity as it is preventive and actively stimulates the immune system, but vaccines are not always fully protective (192) or available. Therefore, there is a need for innovation in veterinary treatment strategies (193) and elucidating NAb and their functionality in veterinary species may provide new exciting opportunities. NAb have been described in veterinary species and it has been demonstrated that they are able to be modified, but the clinical relevance of NAb in veterinary species remains enigmatic. Humans and veterinary species would mutually benefit from the combined effort to study NAb and allow for the reciprocal exchange of findings from their respective fields.

## Intravenous or Oral Administration of Immunoglobulins

Intravenous immunoglobulin (IVIg) preparations contain large amounts of immunoglobulins reactive with various constituents and a portion of these are most likely (self-binding) NAb. IVIg has been used in humans as a therapeutic in immunodeficiency to replace missing immunoglobulins (194). IVIg was used as a successful treatment for Kawasaki disease, which is a pediatric disorder that leads to inflammation of coronary arteries, and diminished coronary dilation and improved coronary flow (195). IVIg was also used as a therapeutic for unexplained recurrent spontaneous abortion, and is especially effective when repetitive miscarriage occurs after an initial live birth (196), suggesting that tolerance against the neonate is breached during first pregnancy and that IVIg, which likely includes NAb, might restore this. This inspires the investigation of NAb-exclusive IVIg administration for the treatment of immune mediated diseases. Natural antibodies, present in IVIG, could be used to prevent autoimmune reactions and to enhance the immune response to vaccination.

Albeit no IVIg *sensu stricto*, intravenous administration of KLH binding NAb to chickens enhanced specific antibody responses to KLH after immunization indicating an “adjuvant” role of NAb (22). Oral administration of NAb could also be beneficial, as pigs fed with pig plasma-derived natural IgG showed a decrease in shedding of *Salmonella enterica diarizone* and three strains of *E. coli* (O138, O149:F4 and F18), and a restoration of microbiota diversity compared to untreated pigs (197). NAb binding glutamate dehydrogenase, carbonic anhydrase, myosin and transferrin were found in unborn calves prior to intake of colostrum, and were greatly enhanced by colostrum resident NAb against the same self-antigens (51). This is in line with previously mentioned findings on the presence of self-binding natural sIgA in colostrum and milk of humans (175–178), speculating that oral ingestion of NAb may lead to immune education and therefore adequate NAb-levels in the neonate. These findings suggest that NAb-levels in neonates, and immunity in general, heavily rely on these early maternal NAb and stresses the importance of breastfeeding or oral Ig-supplementation.

From a veterinary perspective: IVIg procedures are likely not useful, but providence of colostrum and allowing food animals such as calves and piglets to stay with their mothers for an extended period of time would give them a more extensive immune-education that would prevent disease later in life.

## Identifying Natural Antibodies and Target Epitopes to Develop Therapeutic Immunoglobulins

Several IgM NAb-clones were isolated from cancer patients and were able to bind carbohydrate structures on tumors and subsequently decrease tumor burden (136). Another example of an isolated NAb is the IgM clone “EO6,” which was isolated from apolipoprotein E-deficient mice (198). EO6 bound to oxLDL, apoptotic cells, atherosclerotic lesions and oxidized phospholipids whereas it did not recognize native lipoproteins (199). Furthermore, EO6 administration in ApoE deficient mice lead to less oxLDL uptake by macrophages and thus decreased formation of foam cells (200). Intravenous administration of a specific MDA antibody *in vivo* neutralized endogenously generated MDA epitopes that resulted in decreased hepatic inflammation in low-density lipoprotein receptor-deficient mice on a Western-type diet (125).

There is an opportunity to isolate and develop monoclonal therapeutic NAb. This approach would have several benefits in comparison to monoclonal conventional antibodies: (i) NAb would be cost-efficient as they could be directly isolated from donor volunteers which would leave the immunization of mice and other laboratory animals unnecessary. (ii) NAb have been demonstrated to be oligo-specific, so by binding to multiple antigens a single therapeutic NAb could be applied in the treatment of multiple diseases. (iii) NAb that have been investigated so far did not show to bind to healthy tissue or native forms of their target antigens, suggesting less therapeutic side-effects. These therapeutic NAb-inspired immunoglobulins could also be administered to veterinary species to treat inflammatory diseases or prevent cancers, such as Marek's disease in poultry.

## Immunization or Environmental Exposure as Triggers for Natural Antibody Secretion

NAb in neonates have not been positively associated with vaccinations due to maternal IgG. IgG in humans and apes is the only isotype that can pass the placental wall and serves as a single dose of immunoglobulins to the neonate which possess them post-natal up to 12 months. This single dose immunization helps to defend against pathogens in a critical window where the infant's immune system is under development, as demonstrated in agammaglobulinemia patients that were fully protected against bacterial infection up to 6 months after birth (201). While maternal antibodies are considered as essential in the critical window of neonatal immune development, it was demonstrated that maternal IgG may have a substantial inhibitory effect on many human and veterinary vaccines and could even lead to a partial or complete lack of protection in humans and cotton rats [reviewed in (202)].

It can be hypothesized that maternal IgG immunoglobulins are able to neutralize the antigen components from the vaccine and therefore prevent recognition by adaptive immunity. So, the ideal time-point for a vaccination would be when these maternal IgG's have disappeared, but this is highly variable and difficult to predict (202). These effects might be due to neutralization of live vaccines, epitope masking, elimination of antibody-coated vaccines by FcγR-mediated phagocytosis, and inhibition of B-cell activation by Fcγ-receptor mediated signaling. A strategy to evade this phenomenon could be to extend the protection of maternal IgG's and vaccinate with known NAb-epitopes (203), therefore stimulating the development of natural immunity itself and thus provide protection without conventional vaccines. Maternal NAb, likely initiated by the intestinal microbiota, protected neonatal mice in an antigen-non-specific fashion (203). Serum from mice immunized with KLH, DNP and peanut extract showed increased binding of immunoglobulins on brain, liver and spleen slices *in vitro*, demonstrating that NAb-levels can be regulated via (non-specific) immunization (204). This suggests that (non-specific) immunization can increase NAb-levels and therefore be utilized as prevention against autoimmune diseases. In mice solely expressing IgM NAb, approximately 30% of all NAb bound to model oxidation-specific epitopes, atherosclerotic lesions and apoptotic cells. It was hypothesized that these epitopes exert selective pressure to expand NAb, which in turn play an important role in mediating homeostatic functions consequent to inflammation and cell death, as demonstrated by their ability to facilitate apoptotic cell clearance thereby preventing chronic inflammatory diseases and atherosclerosis (71). Indeed, active immunization with phosphorylcholine-enriched pneumococci protected mice against non-alcoholic steatohepatitis (126), whereas immunization with phosphatidylcholine, a component of red blood cells protected mice against Plasmodium infection (165). Also passive immunization of mice with monoclonal IgM against phosphorylcholine reduced vein graft atherosclerosis (128).

Higher IgM NAb-levels, but not IgG, have been found in wild rats compared to their laboratory counterparts (205), suggesting environmental antigens directly influence NAb-levels and diversity. Moreover, several bacterial orders were demonstrated to influence α-Gal NAb-levels (43), while chickens fed with probiotics showed enhanced levels of NAb binding to KLH (174). Recently, it was demonstrated that immunization of rats with model antigens (KLH-FITC or DNP-Ficoll) enhanced the level of antibodies binding various autologous organ extracts for both IgM and IgG, suggesting an enhanced network of NAb (204). Flynn et al. (206) found that domestic cats infected with feline immunodeficiency virus (FIV) showed enhanced levels of antibodies toward non-viral antigens: trinitrophenol (TNP), ovalbumin, beta-galactosidase, and DNA, which were not due to the presence of cross-reacting epitopes on recombinant FIV p17 or p24 antigens and suggesting that B-cell activation associated with infection was polyclonal rather than entirely virus specific (206).

Unpublished results from our lab revealed that chickens kept under high hygienic conditions had low levels of NAb to KLH and self-binding antibodies to liver as opposite to chickens kept under unhygienic conditions. NAb therefore would fit in the hygiene hypothesis, stating that a decreased incidence of infections, especially in the Western world, results in a higher incidence of autoimmunity and allergy (207). Here, microbes would educate natural immunity to peritoneal B1-cells that subsequently secrete homeostatic NAb to prevent autoimmune diseases. Almost by definition, this activity starts immediately after birth and is relevant in early life, precisely as implicated by the hygiene hypothesis. This also would suggest that dietary antigens could influence NAb levels, especially since the “Western Diet” that is rich in refined sugars, salt and saturated fat has been associated with immune alterations, including pathological autoimmunity (208).

Thus, altering antigenic experience by changes in diet or supplementation with probiotics or challenge by microbes might improve NAb-levels and diversity and thus enhance resistance to infection and decrease the incidence of pathological autoimmunity, or enhance the homeostatic function of NAb in preventing mal-behavior and metabolic disorders in veterinary species.

## Breeding or Genetic Modification of Natural Antibodies, Use of Veterinary Species

NAb are often germline encoded, so there is a possibility to modify their levels and diversity on a genetic level. Additionally, it is also important that NAb generally have a restricted V_H_ gene usage, which can also be modified genetically. While performing genetic alterations in humans is obviously difficult due to ethical reasons, veterinary models could be used instead as they are less tightly regulated and experimental circumstances are much more controlled. In addition, breeding companies continuously search for new breeds with higher health status.

There is evidence that breeding for high levels of NAb and NAAb is possible. Different NAAb-levels were earlier determined in inbred mouse strains (78), but studying veterinary species also allows (unexpected) linkage of NAb-levels with various other physiological and important production and welfare traits. The advance of synthetic biology approaches relies on the use of omics information and these greatly improved insights provide opportunities to more closely monitor health conditions, modulate the genetic background, and thus improve animals on a pre-selected genetic background (209). High levels of anti-nuclear immunoglobulins were found to be heritable in sheep and were associated with higher longevity (70). Overall survival during a laying period was higher in chickens with high NAb-levels (129, 130), and life history: “fast” or “slow” correlated with constitutive immune defenses, i.e., that slower developing species showed higher NAb-levels, as was also true for solitary living bird species (210). NAb levels can thus be used to compare constitutive humoral immunity among and within species with respect to strain, age, sex, treatments, ecology, and “life span or history” (33).

Genetic regulation of NAb was demonstrated in poultry that were divergently bred for high levels of anti-KLH NAb (211). Divergent breeding of poultry for NAb also affected their self-antigen binding antibodies (212). A genome wide association study (GWAS) showed that the KLH NAb High line of chickens possessed a single nucleotide polymorphism (SNP) within the TLR1A gene significantly explaining levels of KLH binding IgM's, indicating that TLR1A has a major impact on NAb-levels and/or NAb B-cells. This TLR1A region was also significant for total levels of IgM in blood (163) and most likely levels of IgM antibodies binding self-antigens (213).

NAb-levels to KLH from pigs in high-health environments were proposed to be used as phenotypical predictors for resilience and mortality under a disease challenge, and higher NAb-levels at a young age correspond to increased resilience and decreased mortality in swine (214). NAb against KLH were also found to be heritable in cattle (88, 94). NAb-levels were associated with inflammatory diseases in cattle (215), and NAb-levels in milk and serum correlated both phenotypically and genetically with immune associated traits and diseases in cows (216) including mastitis (131, 217). It is suggested that breeding of cattle against diseases such as mastitis or uterus inflammation may benefit from specifically breeding for high NAb-levels. Different levels of NAb were also found in different genetic lines of common carp (*Cyprinus carpio)* independent of antigen, age and environment, further suggesting that NAb are for an important part under genetic control and could therefore be modulated genetically to improve disease resistance in fish (218) and food animals such as cattle and poultry.

Genetic modulation of NAb could give new insights in key genes that regulate NAb-levels and findings from these studies might be translated to humans in future gene therapies and could possibly restore defected genes associated with decreased NAb-levels that are correlated with disease.

## Concluding Remarks

While Burnet's forbidden clone paradigm still provides a barrier to many immunologists, others gradually accept the existence of NAb and their importance in health and disease. In humans and mice, various infectious, neurological-, tumor-, cardiovascular-, and metabolic diseases were related with (usually decreased) levels of (self-binding) NAb. Still, homeostatic NAb and their target antigens deserve more attention, especially in food animals as they most likely contribute to maintaining health by preventing development of disease in animals as well. While IgM and IgG have been thoroughly investigated in many species, the data on IgA NAb are lacking far behind and should be more intensively investigated. Importantly, NAb may not function completely in an antigen-non-specific manner as previously thought, since relations between diseases, specific antigenic epitopes and specific NAb-isotypes and idiotypes become more apparent. Genomics, proteomics, and quantitative Western blotting approaches will likely reveal many (un)expected self, non-self- and neo-antigens that contribute to the formation and maintenance of NAb. Understanding the functional relationship between NAb and their antigen will lead to intervention, such as vaccination and diet modulation in both humans and animals, or selective breeding and hygiene management strategies in animals. This may result in new health management strategies, such as vaccination and diet modulation in both humans and animals, or selective breeding and hygiene management strategies in animals. Albeit that the role of NAb in veterinary species in contrast to humans is largely unknown, veterinary animals would provide excellent models to investigate the possibilities of modulating NAb, allowing the reciprocal exchange of data that will mutually benefit both human and veterinary immunology.

## Author Contributions

All authors listed have made a substantial, direct and intellectual contribution to the work, and approved it for publication.

## Conflict of Interest

The authors declare that the research was conducted in the absence of any commercial or financial relationships that could be construed as a potential conflict of interest.
